# A High-Sensitivity MEMS Piezoresistive Pressure Sensor for Intracranial Pressure Monitoring

**DOI:** 10.3390/mi17020245

**Published:** 2026-02-13

**Authors:** Zhiwen Yang, Yue Tang, Fang Tang, Bo Xie, Xi Ran, Huikai Xie

**Affiliations:** 1 School of Integrated Circuits and Electronics, Beijing Institute of Technology, Beijing 100081, China; 3220231962@bit.edu.cn (Z.Y.); tangyue@bit.edu.cn (Y.T.); 3220231964@bit.edu.cn (B.X.); 2 State Key Laboratory of Chips and Systems for Advanced Light Field Display, School of Integrated Circuits and Electronics, Beijing Institute of Technology, Beijing 100081, China; 3 Chongqing Institute of Microelectronics and Microsystems, Beijing Institute of Technology, Chongqing 400030, China; 4 Chongqing Engineering Laboratory of High Performance Integrated Circuits, School of Microelectronics and Communication Engineering, Chongqing University, Chongqing 401331, China; eefrank@cqu.edu.cn; 5 Central Research Laboratories, Department of Critical Care, Chongqing University, Chongqing General Hospital, Chongqing 401147, China

**Keywords:** piezoresistive pressure sensor, high sensitivity, intracranial pressure

## Abstract

Accurate monitoring of intracranial pressure (ICP) is critical for the diagnosis and management of neurological disorders. Although various ICP sensors have been developed, their sensitivity is often limited, restricting their ability to detect subtle pressure variations. Therefore, there is a pressing need to develop ICP sensors with enhanced sensitivity to improve measurement accuracy and patient outcomes. In this paper, a highly sensitive and precise pressure sensor for intracranial pressure (ICP) monitoring was proposed. Theoretically, the beam-membrane-island structure was introduced and optimized to improve sensitivity and linearity compared to a flat membrane structure. The notches etched at beam end were designed for further improving sensitivity. Experimentally, the designed sensor achieved a sensitivity of 1.59 mV/V//kPa and a nonlinearity of −0.22% F.S. Additionally, the sensor can detect pressure with centimeter water column (cm H_2_O) resolution, making it suitable for ICP monitoring. This technology holds broad application prospects in the field of medical devices.

## 1. Introduction

Intracranial pressure (ICP) is recognized as one of the most critical physiological parameters for patients suffering from stroke or traumatic brain injury (TBI). Sustained elevation of ICP can lead to secondary brain damage by reducing cerebral blood flow, consequently increasing morbidity and mortality rates. Therefore, the management and continuous monitoring of elevated ICP have long been a major focus in the treatment of neurosurgical patients under critical care [1]. The intracranial compartment primarily consists of the central nervous system (CNS) parenchyma, cerebrospinal fluid (CSF), and arterial and venous blood. According to the Monro–Kellie doctrine, the total intracranial volume, which is the sum of these components, remains constant [2]. Thus, any increase in the volume of one component—such as due to hemorrhage, tumor growth, or edema—must be offset by a corresponding decrease in another component to maintain equilibrium. Measurement of ICP therefore provides vital information about the physiological consequences of intracranial volume variations. Implantable pressure sensors have already been developed and clinically implemented for hydrocephalic patients [3,4,5,6,7]. As a result, ICP monitoring has become an indispensable component of modern neuromonitoring practices [8].

**Figure 1:**
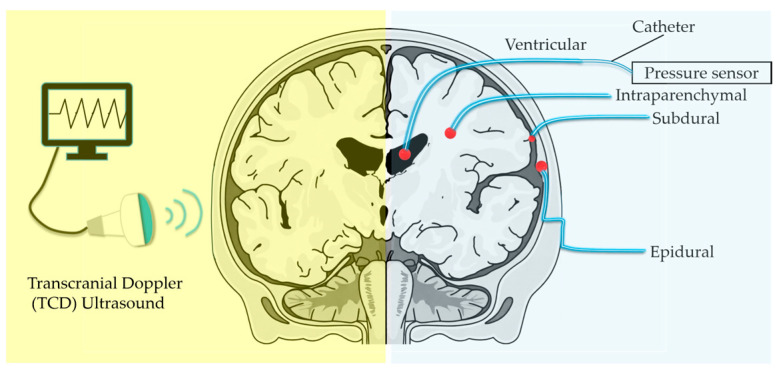
Representative methods of invasive and non-invasive methods for ICP monitoring.

In modern clinical practice, intracranial pressure (ICP) measurement methods can be broadly classified into invasive and non-invasive techniques. Figure 1 illustrates several representative invasive and non-invasive ICP detection methods. The image in the blue area shows that ICP is measured via a ventricular catheter placed within the cerebral ventricles, a brain parenchymal (intraparenchymal) catheter, and ICP sensors placed in the subdural or epidural spaces. The pressure sensor is placed at the catheter tip to measure cerebrospinal fluid (CSF) pressure. The image in the yellow area shows the Transcranial Doppler, a non-invasive tool that measures cerebral blood flow velocity (specifically in the Middle Cerebral Artery) to indirectly estimate ICP. Although non-invasive monitoring eliminates the risks associated with surgical intervention and sensor implantation, its limited accuracy and reliability have hindered its adoption in critical clinical settings [9]. For example, the goal of non-invasive ICP measurement might be to determine whether ICP is above a certain level (e.g., 30~50 mmHg). Measurement of ICP through a cerebrospinal fluid (CSF) ventricular drain remains widely regarded as the clinical gold standard [10]. In recent years, several minimally invasive monitoring systems based on microelectromechanical systems (MEMS) technology have been developed. Typically, a catheter-tip transducer is implanted into the brain parenchyma for the direct detection of intracranial pressure. The MEMS ICP sensor reported by Yanhang Zhang et al. features an ultra-compact size of 150 μm × 150 μm, yet its sensitivity is limited to 12.57 mV/V/MPa [11]. In the work by S.H. Abdul Rahman et al., the introduction of a slotted diaphragm enhanced the mechanical sensitivity of their MEMS ICP sensing device, although the corresponding voltage sensitivity was not reported or determined in the study [12]. Preedipat Sattayasoonthorn et al. presented an ICP monitoring sensor fabricated from liquid crystal polymers (LCP), which exhibits a broad pressure detection range of 0~60.12 mmHg and favorable sensitivity, while the standalone pressure-sensing outer membrane occupies a relatively large volume of 8000 μm × 8000 μm × 100 μm [13,14]. These typically employ catheter-tip transducers placed in contact with brain parenchyma to sense pressure variations. However, the sensitivity of such minimally invasive devices, typically reported to be 0.01 ~ 0.2 mV/V/kPa, remains limited, thereby restricting their ability to resolve subtle intracranial pressure fluctuations compared with conventional invasive sensors [15,16,17,18].

For the piezoresistive sensor, the performance of the sensor is determined by the maximum stress and linearity outputs [19]. A structure with sensitive beams presents its advantages in terms of high sensitivity and high overload resistance compared with the conventional flat diaphragm structures [19,20,21]. The addition of the multi-islands enhances the stiffness of the diaphragm and reduces its deflection [22]. Given the stringent precision requirements of intracranial pressure monitoring, this study presents the design and development of a high-sensitivity piezoresistive MEMS pressure sensor optimized for ICP measurement applications.

## 2. Design Methods of Piezoresistive Sensor

**Figure 2:**
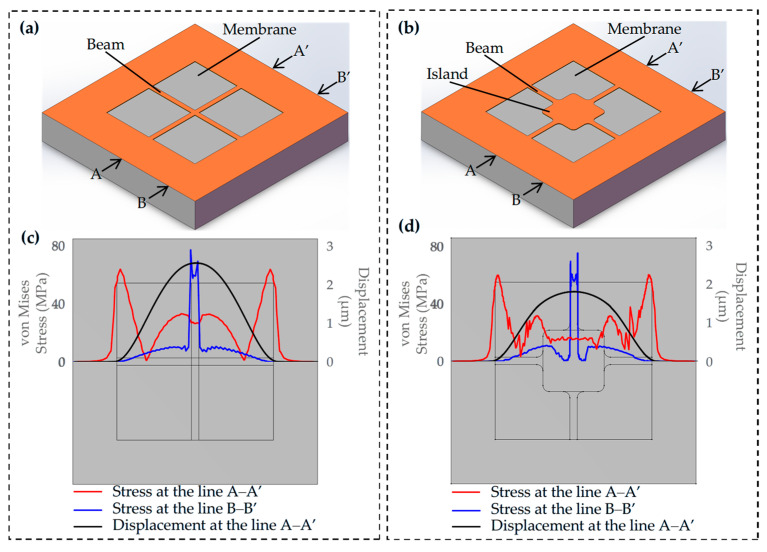
(**a**) Structure of sensor without island; (**b**) structure of sensor with island; (**c**,**d**) simulated stress distribution and displacement of the membrane.

The diaphragm design follows the principle of maximizing sensitivity while preserving high linearity [23]. In this work, a square membrane integrated with four narrow beams and a central mass (as illustrated in Figure 2b) is proposed for sensing pressures below 10 kPa. The beams and the central mass are structurally coupled to form a single integrated system. For the initial sensor design presented in this work, numerical simulation was carried out using COMSOL Multiphysics 6.3, which is consistent with the simulation approach employed in the following investigations. The simulation setup is established based on the finite element method, and the key details are specified as follows: the Solid Mechanics interface is utilized to characterize the mechanical deformation behavior of the sensor structure, with appropriate boundary conditions and loadings applied to simulate the actual operating environment. A structured meshing strategy is adopted to guarantee computational accuracy and efficiency, and the solver is configured with strict convergence criteria to obtain reliable simulation results. This unified simulation protocol ensures the comparability of the results between the initial design and the subsequent optimized configurations. The simulation results are shown in Figure 2.

Figure 2c,d illustrates the simulated distribution of normal stress and displacement within the proposed structure under an applied pressure of 10 kPa. As observed, the stress is mainly concentrated in the beam regions and along the edges of the membrane. The maximum stress of the diaphragm without mass blocks is 76.9 MPa, whereas that of the diaphragm incorporating mass blocks is 75.3 MPa, corresponding to a slight reduction of 2.08%. The maximum displacement of the diaphragm without mass blocks is 2.56 μm, while that with mass blocks is reduced to 1.81 μm, indicating a 29.30% decrease. This notable reduction in deflection demonstrates that the incorporation of mass blocks effectively enhances the mechanical rigidity of the diaphragm, thereby improving the structural stability of the sensor under applied pressure. The central mass positioned on the membrane is strategically designed to optimize the sensor’s mechanical behavior and output linearity. This structure substantially enhances the stiffness of the membrane, which in turn reduces its deflection under external loading. By constraining the membrane deformation within a small, linear elastic range, the design effectively decreases the nonlinearity of the sensing response, ensuring more stable and accurate pressure measurements over the target operating range.

**Figure 3:**
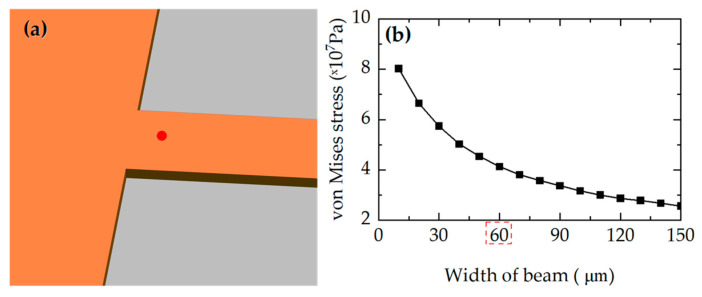
(**a**) The stress extraction points in red at beam end; (**b**) The changes in stress at the red point with the width of the narrow beam.

The effects of beam width are simulated under an applied pressure of 10 kPa, as shown in Figure 3. The beam width varied from 10 μm to 150 μm in increments of 10 μm, and the corresponding variations in stress at the piezoresistors (the red point in Figure 3a) were obtained. The stress decreases significantly as the beam width increases. However, beam width is also determined based on the piezoresistors’ placements. The piezoresistive region requires a width greater than 50 μm. As a result, in this study, the width is selected as 60 µm. At 60 μm, the stress is 41.2 MPa, which is 34.2% higher than the 30.7 MPa observed in the beam-free structure.

**Figure 4:**
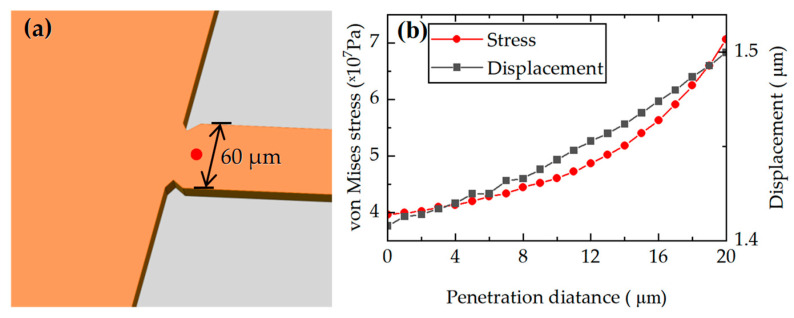
(**a**) The notch on the 60 μm-wide beam and the stress extraction points in red. (**b**) Simulated dimensions of the notch.

A notch is designed at the edge of the 60 μm-wide beam to enhance stress concentration, as illustrated in Figure 4a. The dimensions of the notch are then optimized using COMSOL Multiphysics simulations, as shown in Figure 4b. The results indicate that once the penetration depth reaches 11 μm, the variation in stress becomes more pronounced. Within the 0–11 μm range, the most significant change in stress occurs between 7 μm and 8 μm, and the displacement variation within the aforementioned range is slight. Considering the need to reserve sufficient area for placement of the piezoresistors, the penetration depth is ultimately set to 8 μm.

**Figure 5:**
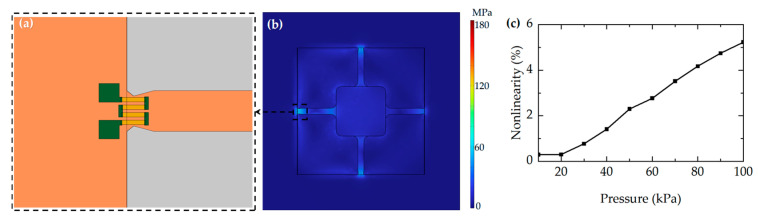
(**a**) The structure of the piezoresistor. (**b**) The stress distribution on the membrane under 10 kPa. (**c**) The nonlinearity of the sensor with different pressures.

A diaphragm featuring four narrow beams and a central mass is derived via simulation analysis of the beam width and the penetration depth of the notch, with the beams incorporating notched corners. The thickness of the diaphragm is 8 μm, while the thickness of the beams and the central mass is 7 μm. The design of piezoresistors on the diaphragm is to be implemented next. The stress distribution at the top of the membrane under an external pressure of 10 kPa is shown in Figure 5b. It can be observed from the figure that stress concentration is highest along the central edges of the membrane. Therefore, the piezoresistors should be positioned at the membrane edges to maximize sensitivity. The piezoresistors are positioned at a distance of 15 μm from the backside diaphragm edge. Their precise geometric dimensions are 130 μm in length, 6.5 μm in width, and 1 μm in thickness, which are designed to ensure stable piezoresistive response and good process compatibility. The piezoresistor is divided into four strips, as illustrated the orange area in Figure 5a. After determining the sensor parameters, the nonlinearity of the sensor under different pressures was simulated. The results are shown in Figure 5c. The applied pressure increases from 10 kPa to 100 kPa in steps of 10 kPa. With increasing pressure, the nonlinearity increases significantly and the linearity gradually deteriorates.

**Figure 6:**
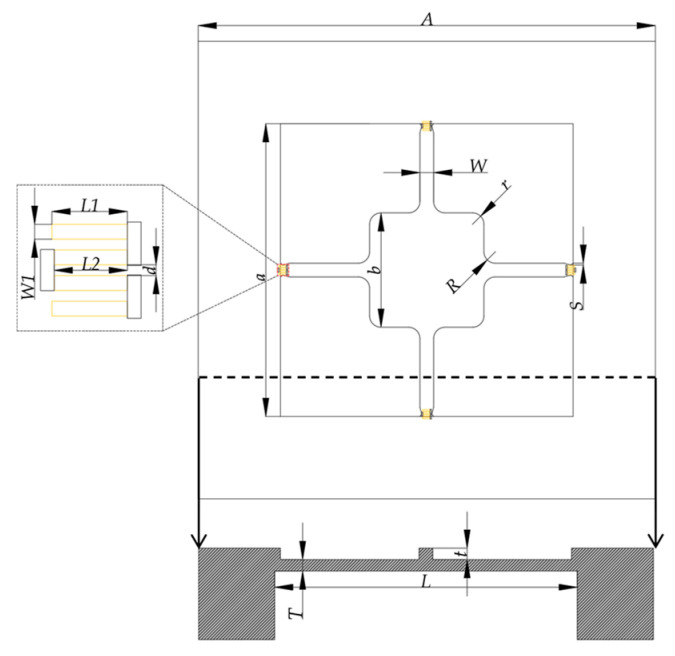
Structural design and parameter schematic of the piezoresistive pressure sensor.

To facilitate a clear understanding of the sensor design, we annotated key structural dimensions in Figure 6, including the front structure, the cross-sectional structure of the device and the structure of piezoresistors. The sensor’s key design parameters are listed in Table 1. The yellow areas are the piezoresistors.

## 3. Fabrication

To achieve the desired gauge resistance, boron implantation at a dose of 5 × 10^14^ cm^−2^ was performed, followed by a post-annealing process at 1000 °C for 30 min to activate the boron ions and to relieve the stress induced during ion implantation. A deep reactive ion etching (DRIE) process was employed to fabricate the membrane, while the beam-narrow-membrane regions were etched using standard reactive ion etching (RIE).

**Figure 7:**
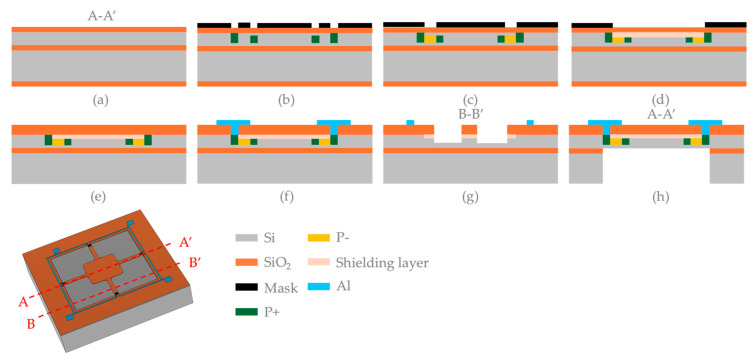
Fabrication process of the proposed sensor: (**a**) Thermal oxidation; (**b**) Heavily doped regions formation; (**c**) Boron ion implantation for piezoresistors; (**d**) Phosphorus ion implantation; (**e**) Silicon dioxide deposition; (**f**) Contact holes etching and Al metallization; (**g**) Front side etching for beam-island-membrane structure; (**h**) Backside etching for cavity formation.

Figure 7 illustrates the fabrication process of the silicon pressure sensor. A 4″ n-type (100) SOI wafer with a resistivity of 1–10 Ω·cm was used. The thicknesses of the top silicon layer and the SiO_2_ layer on the SOI wafer were 15 μm and 1 μm, respectively. Firstly, SiO_2_ layers with a thickness of 20 nm were grown on both sides of the substrate via thermal oxidation (Figure 7a). Photolithography was then employed to pattern the heavily doped regions on the front side of the silicon wafer, followed by boron ion implantation at a dose of 1 × 10^16^ cm^−2^ and an energy of 80 keV (Figure 7b), This was followed by light boron ion diffusion to form the piezoresistors, with a dose of 5 × 10^14^ cm^−2^ and energy 160 keV (Figure 7c). The piezoresistors are arranged within the diaphragm at a junction depth of 1 μm from the top surface. The boron concentration is 3 × 10^18^ cm^−3^. Subsequently, a shielding layer was formed via phosphorus ion implantation at a dose of 1.4 × 10^14^ cm^−2^ and an energy of 37 keV to prevent interference from the external environment (Figure 7d). To electrically activate the boron ions and achieve uniform dopant distribution, the wafer underwent annealing at 1000 °C for 30 min under a nitrogen atmosphere. The SiO_2_ layer was then etched using BOE, and passivation layers of SiO_2_ with a thickness of 150 nm were deposited successively via plasma-enhanced chemical vapor deposition (Figure 7e). Vias were etched using reactive ion etching (RIE), followed by deposition of Al/Ti layers (5000 Å/200 Å) through evaporation to form metal electrodes and establish electrical connections, which were patterned using photolithography and lift-off techniques (Figure 7f). Ohmic contacts between the Al wires and piezoresistors were reinforced through a sintering process. Dry etching of the silicon was employed to form the beam-island-membrane structure with a total etched thickness of 7 μm. The critical sensitive membrane follows a square-shaped design with a side length of 1.28 μm, and a thickness of 8 μm (Figure 7g). Finally, DRIE was used to create a cavity on the back side of the wafer (Figure 7h).

**Figure 8:**
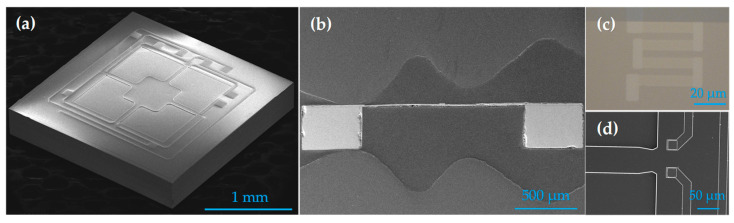
Photographs of the fabricated piezoresistive pressure sensor: (**a**) The SEM image of the overall chip. (**b**) Cross-sectional SEM image of the sensor chip. (**c**) Close-up microscope image of the piezoresistor. (**d**) Close-up SEM image of the notch.

The fabricated sensor die is shown in Figure 8. SEM characterization was performed on the fabricated chips, including both the SEM image of the overall chip (Figure 8a) and cross-sectional images (Figure 8b). The dimensions of the sensor are 2.3 mm × 2.3 mm × 0.37 mm. The results indicate that the sidewalls of the back cavity and the underlying substrate are highly flat and well-defined. In Figure 8c, the microscope image clearly shows the structure of the resistors. The close-up SEM image of the notch is shown in Figure 8d.

## 4. Discussion

### 4.1. Characterization with Gas Pressure

**Figure 9:**
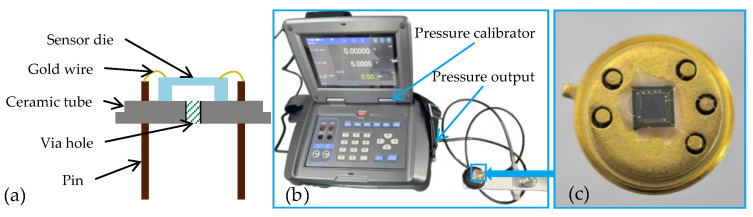
(**a**,**c**) Package of the proposed sensor and (**b**) experimental setup for testing.

As shown in Figure 9, the fabricated pressure sensor was packaged in a TO package with sealant for experimental testing. The sensor die was mounted on a ceramic base, and electrical connections between the sensor pads and the pins were established using gold wires. A through-hole is located at the center of the metal base to allow the application of different reference pressures. The sensor is fixed within the mounting structure, with its pins connected to signal wires routed from the backside of the assembly. A Const811A pressure calibrator was used to apply pressure to the sensor through the pressure port. A constant DC voltage of 5 V was applied to the Wheatstone bridge, and the output response of the sensor was measured over a pressure range from 0 to 10 kPa.

**Figure 10:**
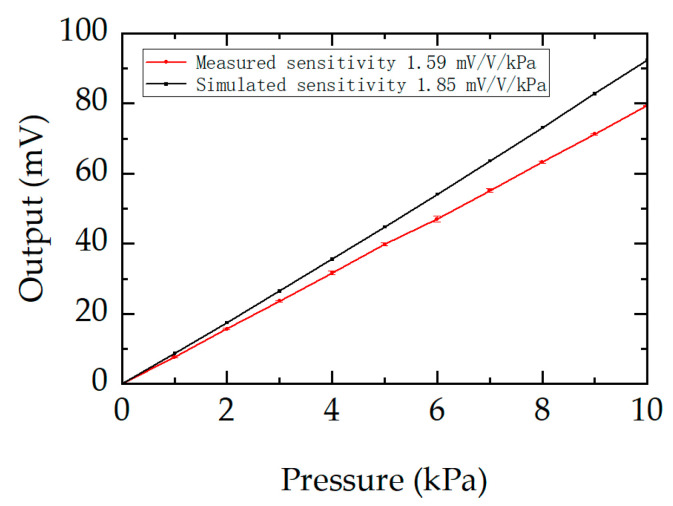
Comparison of measured and simulated sensor outputs.

The sensitivity and nonlinearity of the tested sensor are presented in Figure 10. The experiments were conducted in two processes: a forward process, where the pressure increased from 0 to 10 kPa, and a reverse process, where the pressure decreased from 10 to 0 kPa. In both processes, the pressure step size was 1 kPa. The measured sensitivity of the sensor was 1.59 mV/V/kPa, compared to the simulated value of 1.85 mV/V/kPa. The nonlinearity errors were evaluated using the end-point straight-line method, with the maximum measured nonlinearity being −0.22% F.S. Repeatability is a critical performance indicator for practical sensor applications, and the test results are summarized in Table 2. Three repeated measurements of the voltage–pressure curve of the sensor were performed to obtain the repeatability of the sensor calculated by the Bessel formula.

Finally, the detailed technical characteristics of the fabricated sensor are listed in Table 2.

In conclusion, the proposed sensor maximizes the piezoresistive effect while achieving an optimal balance between sensitivity and linearity. The excellent performance of this chip demonstrates its suitability for intracranial pressure monitoring applications.

### 4.2. Characterization with Liquid Pressure

**Figure 11:**
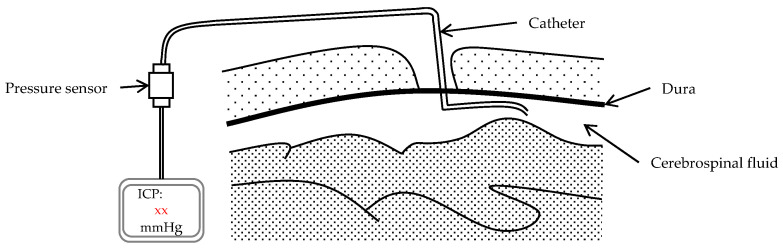
The schematic of the ICP sensor.

A draft of the measurement of ICP through a cerebrospinal fluid (CSF) ventricular drain is shown in Figure 11. The catheter is inserted with its distal tip placed inside the cerebral ventricle, while the proximal end is attached to a pressure sensor. Cerebrospinal fluid is directed into the sensor, thereby facilitating the real-time monitoring of intracranial pressure.

**Figure 12:**
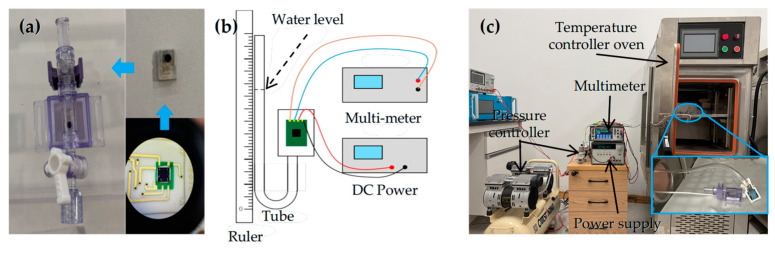
(**a**) The packaged sensor and (**b**) the experimental setup of pressure measurement. (**c**) The thermal experimental setup of pressure measurement.

For intracranial pressure monitoring applications, the sensor chip is packaged as shown in Figure 12a. The sensor is placed inside a tube and electrically connected to the PCB via wire bonding. The sensor was powered by a DC power supply of 5 V. The experimental setup for pressure measurement is illustrated in Figure 12b. The packaged piezoresistive pressure sensor was tested over a temperature range from 0 °C to 120 °C in 20 °C increments. The thermal pressure measurement setup is shown in Figure 12c. At each temperature, the applied pressure varied from 0 to 80 mmHg (equivalent to 0 ~ 10.67 kPa); the pressure step size was 10 mmHg (equivalent to 1.33 kPa).

**Figure 13:**
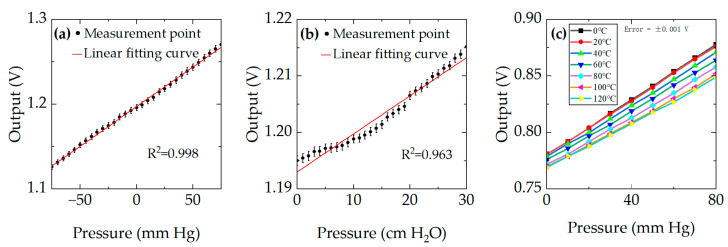
The outputs of experiment: (**a**) the output of chip with pressure from −75 mmHg to 75 mmHg (equivalent to −10 ~ 10 kPa); (**b**) the output of the chip’s detection accuracy; (**c**) the output of the chip at with temperature from 0 °C to 120 °C.

For safety reasons, water was used instead of mercury to test the sensor output under pressures ranging from −75 mmHg to 75 mmHg (equivalent to −10~10 kPa); the pressure step size was 5 mmHg (equivalent to 0.667 kPa). The results are shown in Figure 13a, with a measured sensitivity of 0.996 mV/mmHg. From the fitting curve, it can be seen that there is a good linear relationship (R^2^ = 0.998) between pressure and the output of the sensor. Further experiments were conducted to evaluate the chip’s detection resolution by measuring pressure changes of 1 cm H_2_O (equivalent to 0.0981 kPa). As depicted in Figure 13b, the sensor’s response characteristics were tested and analyzed over a pressure range of 0 to 30 cm H_2_O (equivalent to 0~2.94 kPa). The results indicate that the sensor can reliably detect pressure variations with a resolution of 1 cm H_2_O (equivalent to 0.0981 kPa). From the fitting curve, it can be seen that there is a linear relationship (R^2^ = 0.963) between pressure and the output of the sensor. The results of the thermal pressure measurement are shown in Figure 13c. For each temperature, the sensor output voltage increases nearly linearly with applied pressure, demonstrating excellent goodness of linear fit. The sensor exhibits an excellent linear relationship over the entire tested temperature range (R^2^ > 0.999 for all temperatures). The maximum calculated Temperature Coefficient of Sensitivity (TCS) is 146 ppm/°C, and the maximum calculated Temperature Coefficient of Zero (TCZ) is 103 ppm/°C.

The comparisons of size, sensitivity, nonlinearity and operating range of the proposed sensor with different piezoresistive pressure sensors are shown in Table 3. All the sensors selected for comparison are designed for intracranial pressure (ICP) monitoring. Although the sensor proposed in this work has a relatively larger device size, its sensitivity and linearity are superior to those of the other comparable sensors.

## 5. Conclusions

This work presents a highly sensitive and overload-resistant pressure sensor based on a beam-membrane-island structure. To validate the proposed design, a prototype was simulated, optimized, and fabricated. Finite element modeling (FEM) was employed to analyze membrane deflection under varying pressures. Experimental results demonstrate the effectiveness of the proposed structure in enhancing sensitivity while maintaining overload tolerance. Specifically, the sensor exhibits high sensitivity and robust overload capacity, making it a promising candidate for intracranial pressure (ICP) monitoring applications.

The fabricated chip is capable of detecting pressure variations with a resolution of 1 cm H_2_O (equivalent to 0.0981 kPa), satisfying the precision requirements for ICP monitoring (normal range: 5~15 mmHg, equivalent to 6.8~20.4 cm H_2_O or 0.667 ~ 2 kPa). The maximum calculated Temperature Coefficient of Sensitivity (TCS) is 146 ppm/°C, and the maximum calculated Temperature Coefficient of Zero (TCZ) is 103 ppm/°C. The proposed sensor possesses extremely high sensitivity under the condition of low nonlinearity. With its balanced sensitivity and satisfactory precision, the sensor emerges as a promising candidate for ICP monitoring applications. Future work will focus on further investigation of the sensor’s static performance through improvements in experimental conditions and fabrication precision. Moreover, comprehensive performance characterization and practical application validation will be implemented to evaluate the feasibility of the sensor in clinical medical pressure detection, which will lay a solid foundation for its engineering popularization.

## Figures and Tables

**Table 1:** Primary design parameters of the pressure sensor.

Parameter	Description	Value (μm)
*A*	The side length of sensor	2300
*a*	The side length of front diaphragm	1280
*b*	The side length of central mass	500
*L*	The side length of backside diaphragm	1330
*W*	The width of the beams	60
*R*	The corner radius	50
*r*	The corner radius of central mass	50
*S*	The penetration distance of notch	8
*t*	The thickness of beams and central mass	7
*T*	The thickness of diaphragm	8
*L1*	The length of single-strip piezoresistors	33
*L2*	The length of the other single-strip piezoresistors	32
*W1*	The width of piezoresistors	6.5
*d*	The distance between two strips of piezoresistors	4.7

**Table 2:** Characteristics of the sensor.

Parameter	Value
Maximum pressure (kPa)	10
Supply voltage (V)	5
Full-scale span (mV)	79.35
Sensitivity (mV/V/kPa)	1.59
Nonlinearity (%F.S)	−0.22
Hysteresis (%F.S)	1.10
Repeatability (%F.S)	2.40

**Table 3:** Comparisons of different piezoresistive ICP sensors.

Parameter	This Work	NOVA-P330	[16]	[17]	[18]
Diaphragm (μm × μm)	1300 × 1300	-	400 × 400	900 × 900	radius 500
Operating range (mmHg)	−75 to 75	−30 to 300	0 to 7500	−75 to 250	0 to 45
Sensitivity (mV/V/kPa)	1.59	0.112	0.127	0.075	1.275
Nonlinearity error (%F.S)	−0.22	0.15	-	<1.2	0.996
Test condition	Gas	Gas	Gas	Gas	Liquid

## Data Availability

Data are available from the authors on request.
